# A Blade Defect Diagnosis Method by Fusing Blade Tip Timing and Tip Clearance Information

**DOI:** 10.3390/s18072166

**Published:** 2018-07-05

**Authors:** Ji-wang Zhang, Lai-bin Zhang, Li-xiang Duan

**Affiliations:** School of Mechanical and Transportation Engineering, China University of Petroleum (Beijing), Beijing 102249, China; jiwangz.cupb.china@gmail.com (J.-w.Z.); zhanglb@cup.edu.cn (L.-b.Z.)

**Keywords:** high-speed blades, blade tip-timing (BTT), tip clearance (TC), under-sampled signal, CNN

## Abstract

Blade tip timing (BTT) technology is considered the most promising method for blade vibration measurements due to the advantages of its simplicity and non-contact measurement capacity. Nevertheless, BTT technology still suffers from two problems, which are (1) the requirements of domain expertise and prior knowledge of BTT signals analysis due to severe under-sampling; and (2) that the traditional BTT method can only judge whether there is a defect in the blade but it cannot judge the severity and the location of the defect. Thus, how to overcome the above drawbacks has become a big challenge. Aiming at under-sampled BTT signals, a feature learning method using a convolutional neural network (CNN) is introduced. In this way, some new fault-sensitive features can be adaptively learned from raw under-sampled data and it is therefore no longer necessary to rely on prior knowledge. At the same time, research has found that tip clearance (TC) is also very sensitive to the blade state, especially regarding defect severity and location. A novel analysis method fusing TC and BTT signals is proposed in this paper. The goal of this approach is to integrate tip clearance information with tip timing information for blade fault detection. The method consists of four key steps: First, we extract the TC and BTT signals from raw pulse data; second, TC statistical features and BTT deep learning features will be extracted and fused using the kernel principal component analysis (KPCA) method; then, model training and selection are carried out; and finally, 16 sets of experiments are carried out to validate the feasibility of the proposed method and the classification accuracy achieves 95%, which is far higher than the traditional diagnostic method.

## 1. Introduction

High-speed blades are core components in turbomachinery such as aircraft engines and steam turbines. They often suffer drastic vibration under centrifugal force and fluid exciting force [1,2]. These vibrations will greatly reduce the residual life and performance of the blades and eventually may lead to catastrophic accidents [3]. How to monitor the blade condition on-line has been a research hotspot in recent years. Among many monitoring methods, blade tip timing is considered to be one of the most promising methods due to the advantages of its ability to make non-contact measurements and its low cost [4,5,6,7]. However, BTT data is always an under-sampled signal due to the restriction of probe quantities [8]. At the same time, the traditional analysis method based on the BTT signal can only judge whether the blade is damaged or not but it is difficult to judge the severity and location of the damage [9]. In addition, the BTT signal is difficult to analyze due to the severe under-sampling [8,10,11,12]. Thus, a problem has been proposed in this paper: how to extract sensitive features from the under-sampled BTT signals to identify blade fault severity and position.

Nowadays, some methods have been proposed to obtain blade vibration responses from under-sampled BTT data. Ref. [7] test the forced response of rotating bladed disks using a BTT data post-processing method called circumferential Fourier fit (CFF). Ref. [13] proposed a single-probe optical sensor method measuring the blade vibration detection of high-speed compressors. Refs. [14,15] introduced an improved single-parameter tip timing method for turbomachinery blade vibration measurements using optical laser probes. Ref. [16] proposed a subspace BTT data reconstruction method to identify the modal parameters of a mistuned bladed disk. Ref. [17] introduced a multisampling BTT data analysis method which achieved the blade vibration measurement. Ref. [18] proposed to investigate blade responses from tip timing data where each blade responds at a single frequency. The blade vibration parameters can be obtained but sufficient prior knowledge is the basis for the application of these methods. Aiming at this problem, Tianjin University [19] proposed a new method called “5 + 2” to extend the ability of the monitoring system. However, it can only identify the blade response within a range of 15 times the rotation frequency. Besides this, some reconstruction algorithms [3,20,21,22] have been proposed to restore the integral vibration signals. However, they are almost unavailable in the working environment due to the influence of noise. Recently, the feature learning method has provided a new idea for BTT signal analysis. As opposed to the traditional methods, feature learning can mine the comprehensive feature representation directly from input data, which does not need prior knowledge and expert experience [23,24,25]. Therefore, a new BTT data analysis method based on deep learning is presented to overcome the difficulties in dealing with under-sampled signals.

Few studies aiming at the problems of blade fault severity and position identification have been found in the open literature. In this paper, the blade vibration response is analyzed using FEM and the result shows that the tip clearance is very sensitive to blade states, including blade vibration, defect severity and location. Therefore, blade states and defects could be monitored and identified by fusing the blade tip timing information and the tip clearance information.

Based on this, the method of fusing BTT and TC signals for the on-line monitoring of high-speed blades using an eddy current sensor is proposed. The main contribution of this method includes two parts. First, a new under-sampled BTT data analysis method based on feature learning is proposed. Second, the blade fault severity and position could be identified by fusing TC and BTT information only using one sensor. The analysis result shows that the proposed method is more effective at identifying the blade states.

The remainder of this paper is organized as follows. Section 2 provides the related work of the BTT method and analyzes the blade vibration response based on FEM. Section 3 describes in detail the proposed method for blade condition identification. In Section 4, the experimental tests are carried out to validate the feasibility of the proposed method. Finally, some major conclusions are summarized in Section 5.

## 2. Related Work

### 2.1. The Principle of Blade Tip Timing

**Figure 1:**
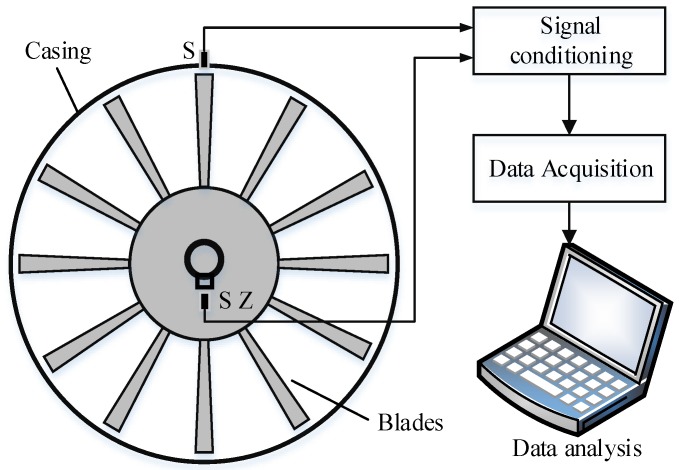
The schematic of the blade tip timing (BTT) system.

The BTT technique is a non-contact blade vibration monitoring method. As shown in Figure 1, the probe (S) is mounted on a stationary casing. The arrival time of the blade pass probe will be recorded by a series of pulse signals. An additional probe, SZ is mounted in front of the shaft as a reference sensor. The arrival time interval is determined by the rotating speed and blade tip radius. Once the blade is damaged, the time interval will be changed and these deviations will be used to analyze the blade condition.

Obviously, the BTT data belongs to the severe under-sampled signal because its sampling frequency is far less than the blade vibration frequency. Thus, domain expertise and prior knowledge is necessary for BTT signals analysis. In addition, the traditional method only judges the blade’s running state depending on whether the blade vibration frequency changes. Therefore, it can only judge whether there is a defect in the blade but it cannot judge the severity and the location of the defect. Therefore, how to extract sensitive features from the under-sampled BTT signals for the identification of blade fault severity and position is still a big challenge for the BTT technique.

### 2.2. Blade Dynamics Analysis

#### 2.2.1. Modal Analysis

**Figure 2:**
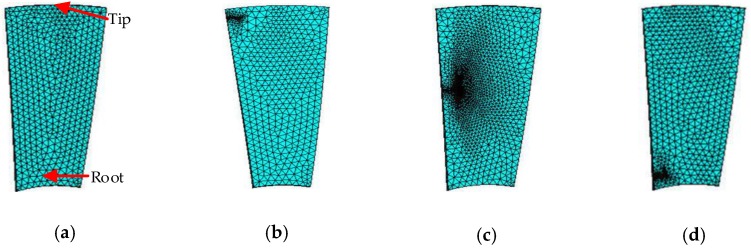
Blade modeling and meshing. (**a**) Flawless state; (**b**) Blade tip crack; (**c**) Blade middle crack; and (**d**) Blade root crack.

In order to master the vibration mode of high-speed rotating blades, finite element analysis (FEA) was performed by using Ansys simulation software. Four kinds of experiments were set up: flawless, tip crack, middle crack and root crack. The blade modeling and meshing (the tetrahedron element) is as shown in Figure 2. The blade parameters are set as shown in Table 1. In practice, the blade root is connected to the hub, so the blade root is fixed and the rest is free. Besides this, the mesh near the crack needs to be densified to obtain accurate simulation results.

**Figure 3:**
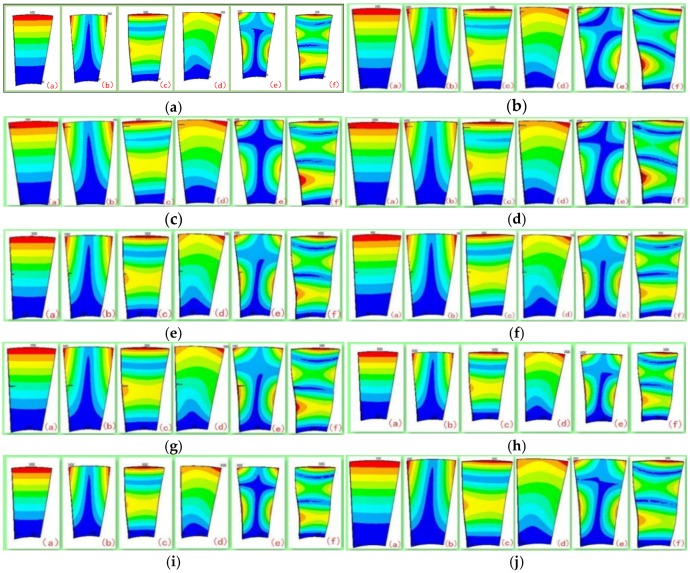
Blade modal shapes. (**a**) Flawless state (N); (**b**) Blade tip crack (Tip 1); (**c**) Blade tip crack (Tip 2); (**d**) Blade tip crack (Tip 3); (**e**) Blade middle crack (Middle 1); (**f**) Blade middle crack (Middle 2); (**g**) Blade middle crack (Middle 3); (**h**) Blade root crack (Root 1); (**i**) Blade root crack (Root 2); and (**j**) Blade root crack (Root 3).

**Figure 4:**
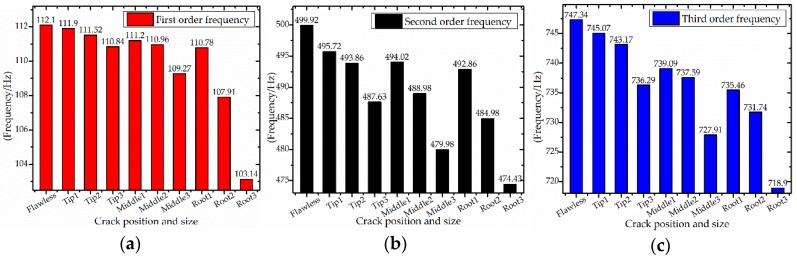
Blade modal frequency. (**a**) First order frequency; (**b**) Second order frequency; (**c**) Third order frequency.

Ten sets of experiments (as shown in Table 2) are carried out and the simulation results are shown in Figure 3. The FEM analysis results showed that the main blade vibration forms were shimmy, torsional vibration and coupled vibration and that the low-order vibration of blade is shimmy. At the same time, the blade modal frequency with different crack locations and crack sizes are also analyzed as shown in Figure 4.

At the same time, the responses of blade vibration are also analyzed. As shown in Figure 4, the blade defect will change the modal frequency. At the same defect position, the modal frequency decreases with the increase of defect size. With the same defect size, blade modal frequencies are more sensitive to root defects. Different positions and sizes of defects have different effects on the modal frequencies of blades. Therefore, the blade vibration frequency can be used as a sensitive parameter to indicate the blade state.

#### 2.2.2. Vibration Response Analysis

**Figure 5:**
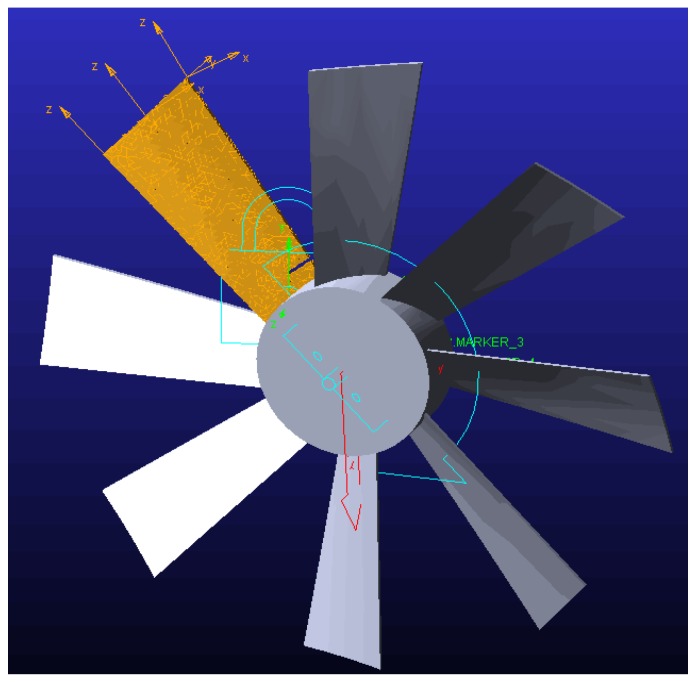
Blade vibration response analysis.

**Figure 6:**
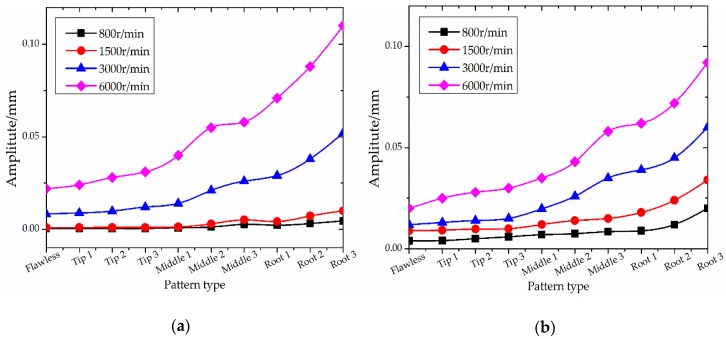
The relationship between blade states and deviation/tip clearance. (**a**) Deviation of blade dynamic balance; (**b**) tip clearances.

Using Adams software. The Adams software has great advantages for multi-body dynamics analysis which have been generally recognized by industry and academia [26]. In this section, the vibration responses of different blade cracks are analyzed. First, a rotating blade model is established by using the cyclic symmetric model of ANSY and the model parameters are set as shown in Table 1. Then, the blades are processed flexibly and the model is imported into Adams for dynamic simulation as shown in Figure 5. Finally, the vibration response signals of the blade tip are extracted and analyzed. Ten sets of experiments (as shown in Table 2) are carried out and the analysis results are shown in Figure 6.

The simulation results show that the blade vibration response varies in different conditions (crack locations, sizes and rotating speeds). With the increase of the crack length and rotating speed, the deviation of blade dynamic balance and tip clearance also increased greatly. That means the tip clearance is very sensitive to blade states. This information could be fused into the BTT data to improve the diagnostic accuracy of the blade status.

### 2.3. Sensors Selection

**Figure 7:**
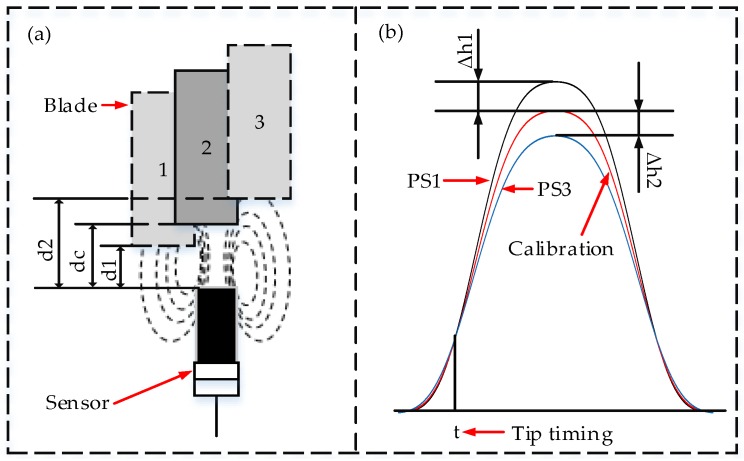
The measurement principle of BTT and tip clearance (TC) signals. (**a**) Schematic diagram of different tip clearances; (**b**) Impulse response signals.

Nowadays, the BTT probes mainly include optical fiber, capacitive and eddy current types [27]. The measuring principle of the optical fiber is to record the reflected light as the arrival time of the blade tip. The optical fiber sensor has a fine resolution and large bandwidth but a clean test environment is needed, which greatly limits its application [28]. The capacitive sensor [29] obtains the blade arrival times by using the change of capacitance between the probe and the blade tip. But the measurement accuracy is susceptible to the influence of the measured media. The measuring principle of the eddy current sensor [30] is based on the electromagnetic induction phenomenon. The probe will generate the feedback voltage when a conductor passes. Compared with the optical fiber and capacitive sensor, the eddy current sensor is widely used for its good sensitivity and strong resistance to interference in actual test. In addition, the TC and BTT signals could be simultaneously measured by one probe. Based on the above advantages, the eddy current sensor will be recommended as a BTT probe in this paper. The principle of tip clearance measurement is as shown in Figure 7 and the different impulse response amplitudes (Figure 7b) will be generated due to different tip clearances (Figure 7a). The ideal tip timing signal is a rectangular pulse signal but actually it is a gradual pulse signal. In Figure 7a, d1, dc and d3 denote different clearances between the blade tip and sensor, where dc represents the calibration clearance value. In Figure 7b, the curves of PS1 and PS3 denote the impulse response signals under different tip clearances of d1 and d3. Δh1 and Δh2 indicate the difference of pulse amplitude due to the difference of tip clearance. The time t indicates the time at which the blade arrives at the sensor (the rising edge of the pulse signal).

### 2.4. Convolutional Neural Network

**Figure 8:**
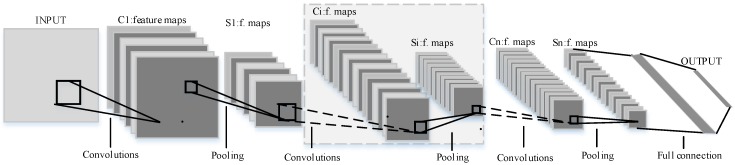
Schematic diagram of the convolutional neural network (CNN).

The convolutional neural network (CNN) is a learning model bio-inspired by an animal visual cortex which tries to learn an optimum set of g kernels relative to a specific task from a dataset [31]. CNN is a multilayer neural network which consists of some filter layers and one classification layer [32]. The filter layer is used to extract features from the input data, which includes four sub-layers: the convolutional layer, batch normalization layer, activation layer and the pooling layer [33]. The classification layer is a multi-layer perceptron. Figure 8 shows the architecture of the CNN.

The feature mining process mainly includes the convolutional layer and pooling layer. The convolutional layer convolves the input from the previous layer with filter kernels and generates a feature output, generally called a feature map. Each filter uses the same kernel to extract the local feature of the input data. One filter corresponds to one frame in the next layer. We use $K_{i}^{l}$ to denote the weights of the *i*-th filter kernel in layer *l* and use $X^{l(r^{j})}$ to denote the *j*-th local region in convolutional layer *l*. Then, the convolution process is described as follows:

(1) $$y^{l(i,j)}=K_{i}^{l}\times X^{l(r^{j})}=\sum_{j^{\prime }=0}^{W}K_{i}^{l}(j^{\prime })X^{l(j+j^{\prime })}$$

A pooling layer usually follows a convolution layer in the CNN architecture. It functions as a down-sampling operation which reduces the feature dimensions. The down-sampling operation can be applied by max pooling, mean pooling or weighted pooling. In these methods, the max-pooling layer is most commonly used in CNN, which performs the local max operation over the input features and obtains location-invariant features. The max-pooling transformation can be described as follows:

(2) $$p^{l(i,j)}=\max_{(j-1)W+1\leq t\leq jW}\left\{a^{{}^{l(i,t)}}\right\}$$

where $a^{l(i,t)}$ denotes the value of *t*-th neuron in the *i*-th frame of layer *l*, t∈[(j−1)W+1,jW], *W* is the width of the pooling region and $p^{l(i,j)}$ denotes the corresponding value of the neuron in layer *l* of the pooling operation.

Through the above process, the local features can be directly learned from raw input data and this method does not need prior knowledge and expert experience.

## 3. Methods

**Figure 9:**
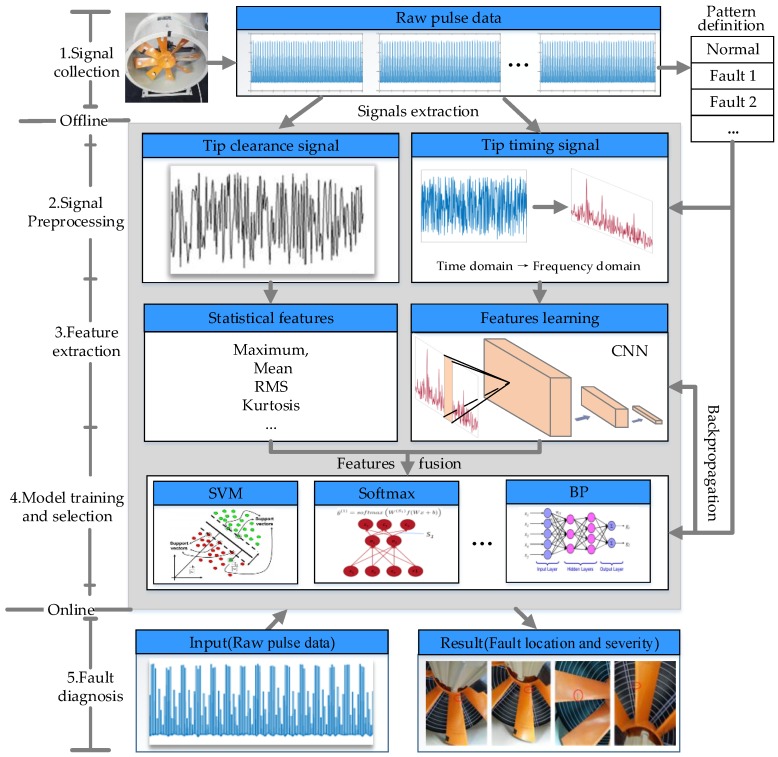
The schematic of the high-speed blades’ online fault diagnosis method.

As we noted before, feature extraction and selection are not trivial tasks for an under-sampled BTT signal. In this paper, we propose a method for automatic feature extraction from an under-sampled signal using a convolutional neural network aiming at the problem of blade fault severity and fault position identification. An on-line monitoring and diagnosis method by fusing blade tip timing and tip clearance information is presented as shown in Figure 9 and is composed of the following steps: (a) signal collection; (b) signal pre-processing; (c) feature extraction and fusion; (d) model training and selection; and (e) online fault diagnosis.

### 3.1. Signal Collection

The main purpose of this step is to acquire raw pulse data under different fault modes and fault severities using an eddy current sensor. The raw data will be normalized to [0,1] in order to facilitate the subsequent analysis.

### 3.2. Signal Pre-Processing

Firstly, the TC and BTT signal are extracted from the raw pulse data. Due to the response frequency of the eddy current sensor being limited, the sampling frequency only meets the requirement of resolution with difficulty. This means that the system will introduce measurement errors. Aiming at this problem, cubic spline interpolation processing is proposed (at least 100 points are inserted between the sampling points), which makes the signal time resolution less than 1 us. At the same time, the raw signal is also affected by noise such as casing vibration and random noise. The outliers and noise should be eliminated by using the wavelet or EEMD (Ensemble Empirical Mode Decomposition) method. Then, we convert the BTT signal from the time domain into frequency domain using fast Fourier transformation (FFT) as a CNN input.

### 3.3. Feature Extraction and Fusion

#### 3.3.1. Statistical Features—TC Signal

The variation of tip clearance is an important index that can indicate the condition of high-speed blades (Section 2.2.2). The study found that the TC signal has the characteristics of randomness and under-sampling and therefore time domain statistical features of the maximum, standard deviation, root amplitude, mean value, mean square root, kurtosis and peak to peak value are extracted for the following analysis. They are calculated by the formulation of Table 3, where *x* (*t*) represents sample signal, *N* represents the sample length of vibration signal, max(*x* (*t*)) and min(*x* (*t*)) represents maximal and minimum value of signal.

#### 3.3.2. Feature Learning—BTT Signal

Unlike the TC signal, the BTT signal is not only under-sampled but also periodic. For periodic signals, frequency features are more sensitive to device running conditions; thus, the time-domain BTT data will be converted into frequency-domain data for further analysis. However, due to the under-sampling of the BTT signal, the fault features and fault modes cannot be matched exactly and some unknown difference frequencies will appear in the frequency-domain signal. How to extract sensitive features from the frequency signal has become the key to blade condition identification. Due to the good data learning ability of the convolutional neural network, we propose the automatic feature learning method for BTT signal by using CNN.

The architecture of the proposed CNN consists of six convolutional layers, a fully-connected layer and a Softmax layer. The whole structure was arranged as follows: ‘‘Input-C1-S1-C2-S2-C3-S3-C4-S4-C5-S5-C6-S6-fully connected-Softmax.” The BTT frequency domain signals will be used as input data and the size of the convolutional kernel is as shown in Table 4. The first kernel size is 64 × 1 and the rest is 3 × 1. Multilayer small convolutional kernels could make the network deeper, which could gain better feature representations and improve the performance of the network. The pooling type is max pooling. The parameters of the proposed network are detailed in Table 4.

#### 3.3.3. Feature Fusion

In order to improve the diagnostic accuracy, the method of fusing BTT and TC information using kernel principal component analysis (KPCA) is proposed. KPCA is a dimension reduction method applied to principal components extraction, which can extract the nonlinear structure information in the data set. More details on KPCA can be found in the provided Refs [34,35,36,37,38]. By using the KPCA method, the main components of the statistical features (TC data) and the learning features (BTT data) will be extracted for later diagnosis.

### 3.4. Model Selection

The appropriate classifier is a prerequisite of good classification results. In Section, 4 typical classifiers (SVM (support vector machine), BP (back propagation neural network), FNN (fuzzy neural network) and Softmax) will be selected as a classification model for subsequent online diagnostics. In order to evaluate the performance of the classifiers, three indexes are introduced: *DR*, *FAR* and *cc*. *DR* denotes the diagnosis rate, FAR denotes the false alarm rate and *cc* represents the correlation between the analysis result and the actual situation, ranging from −1 to 1, where 1 implies that the analysis result is fully consistent with the actual situation and −1 means the analysis result is random. The corresponding formulas are shown in Equations (3)–(5).

(3) $$DR=\frac{TN}{TN+FR}$$

(4) $$FAR=\frac{FN}{FN+TP}$$

(5) $$cc=\frac{TP\times TN-FP\times FN}{\sqrt{\left(TP+FN)(TP+FP)(TN+FP)(TN+FN\right)}}$$

where *TP* denotes that the normal behavior is correctly forecasted, *FP* indicates that the abnormal behavior is judged as normal, *FN* denotes that the normal behavior is wrongly thought as abnormal and *TN* represents that the abnormal behavior is correctly diagnosed.

### 3.5. Online Fault Diagnosis

After the models have been trained using the previous method, on-line tests could be performed. We input new fusion features from online collection data into the obtained models without changing the inner parameters. The diagnostic system will output two results: fault location and fault severity.

## 4. Experiments

### 4.1. Experimental Set-Up

**Figure 10:**
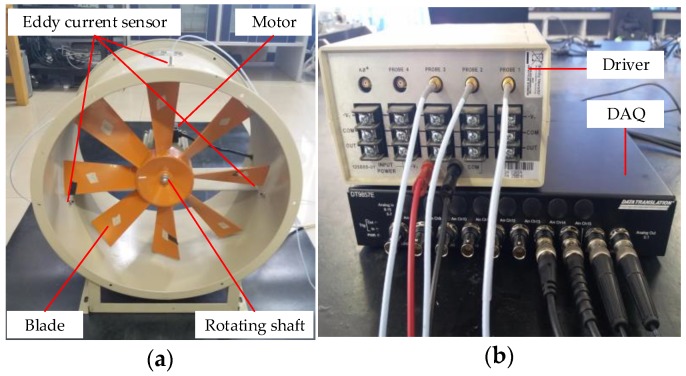
Test rig. (**a**) Major structure; (**b**) Auxiliary structure.

An experimental set-up similar to Figure 4 is built to validate the feasibility of the proposed method, shown in Figure 10. Three eddy current probes are uniformly embedded in the circular casing and simultaneously active to collect arrival times and tip clearances and the detailed experimental parameters are shown in Table 1 (Section 2.2). Table 5 summarizes the fault configurations and severity of the rotating blades. The raw impulse response signals are collected by eddy current sensors (Bently, 330-903-000-310-020-0) with the 20 kHz sampling. The probe diameter is 5 mm, the test range is 0.2 to 2.3 mm, the resolution is 7.87 V/mm and the bandwidth is 0~20 kHz. The driving frequency of driver is 1 MHz. The outputs of the sensors are fed into a laptop through a data acquisition box (DAQ, DT9857E). The experimental procedure is shown as follows. Firstly, the TC and BTT data of the health blade are collected. Then, the damage tests are carried out in accordance with the crack location and crack size as shown by the test number in Table 5. In the test, the 1.5 mm tip clearance is taken as the reference value to measure the TC information.

### 4.2. Classifier Selection

The four classifiers SVM, BP, Softmax and FNN are compared using introduced indexes in Section 3.4 and the performance of the classifiers is shown in Table 6. TrD denotes the training time and TeD denotes the test time. The experiment was processed within a MATLAB R2016b environment, which was running on a PC powered by an Intel Core i5 CPU and 3.0 GB RAM.

As seen in Table 6, among the four classifiers, the SVM has the highest accuracy (94.5%) and the largest correlation coefficient (0.951). The FAR of SVM is also minimal, at only 9%. We can also see that the SVM has obvious advantages in training time and test time over the others. Therefore, the SVM will be chosen as a classification model for subsequent online diagnostics.

### 4.3. Result and Discussion

**Figure 11:**
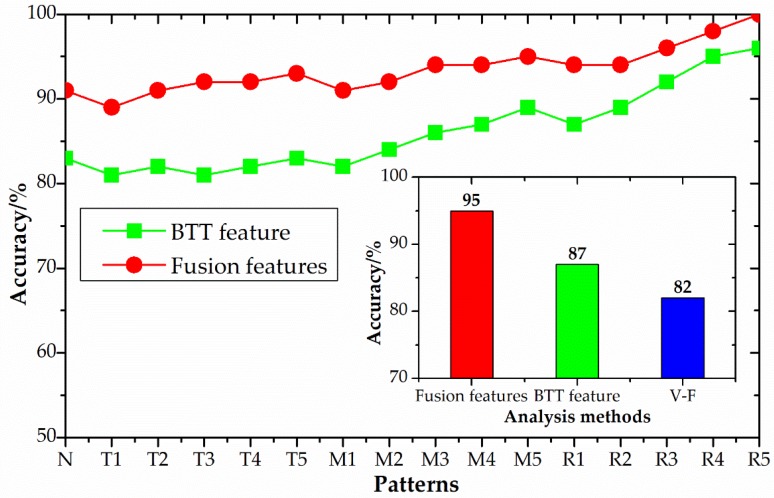
Comparison of diagnostic results with different methods.

With the experimental configuration shown in Table 1, 16 patterns with different crack locations and crack sizes were performed. In order to ensure the accuracy of the test results, a repeated and long-time continuous testing is done. A total of 150 sets of data were collated and divided into two parts: 50 sets of training samples and 100 sets of testing samples. Other detailed experimental parameters are shown in Table 4 and Table 5. The test results using the proposed method are shown in Table 7 and the comparison of diagnostic results based on fusion features, BTT features (extracted from the BTT frequency domain signals only using CNN) and the traditional vibration frequency (V-F) method is shown in Figure 11. The traditional V-F method can only judge whether there is a fault but it cannot judge the location and severity of the fault. Thus, the result of V-F analysis is only the total value and not the result curve.

As seen in Figure 11, the diagnosis accuracy based on fusion features is obviously higher than BTT features and the traditional method, especially for tip crack patterns. The diagnostic accuracies of fusion features, BTT features and the traditional vibration frequency method were 95%, 87% and 82%, respectively. The traditional vibration frequency analysis method can only judge whether there is a fault but it cannot judge the location and severity of the fault. The BTT feature method has a low identification accuracy. This is because it fails to adequately capture valid information that can express failure patterns. The proposed method of fusing BTT and TC information based on a convolutional neural network can not only improve the diagnostic accuracy but also judge the location and severity of the fault. In summary, we can conclude that the proposed method is more sensitive and accurate at identifying high-speed blade conditions.

However, it is important to note that the trained model is only applicable to the same blades under the same operation conditions. Therefore, once the blade types and working conditions are changed, the model needs to be retrained. It should also be noted that sufficient training data is necessary to avoid over-fitting in model training.

## 5. Conclusions

This paper discussed the shortcomings of the blade monitoring system using BTT technology, including its poor diagnostic accuracy and failure to recognize fault location and severity. At the same time, the vibration characteristics of a high-speed blade and under-sampled BTT signal processing based on a convolutional neural network are studied in detail. Finally, the proposed method is verified by experiments. The main conclusions and contributions can be summarized as follows.

1. The paper studied the dynamics of high-speed blades, including through modal analysis and vibration response analysis. The result showed that the blade vibration form mainly includes shimmy, torsional vibration and coupled vibrations. Besides this, the tip clearance was very sensitive to blade states.
2. The eddy current sensor has been recommended as the blade tip timing probe, which achieved the simultaneous acquisition of BTT and TC signals only using one probe.
3. A new under-sampled BTT data analysis method based on a convolutional neural network is introduced. In this way, some new fault-sensitive features can be adaptively learned from raw data and no longer rely on prior knowledge.
4. An information fusion method based on statistical features and learning features has been introduced for high-speed blade on-line monitoring and diagnosis using BTT and TC signals. Compared with the traditional method, this method yielded higher diagnostic accuracy.

Future work will focus on the characteristics of blade vibration under actual conditions, including shafting faults, variable speeds and variable loads. Additionally, further research on how to improve the sampling frequency of the eddy current sensor is still needed.

## Figures and Tables

**Table 1:** Blade parameters.

Parameters	Material	Blade Length	Width (Top/Root)	Thickness	Poisson’s Ratio
**Properties**	Aluminum 7075	175 mm	84 mm/58 mm	2.5 mm	0.33

**Table 2:** Fault pattern of the blades.

No.	Fault Patterns	Crack Positions	Crack (mm)	Percentage
1	N	None	0.0	0
2	Tip 1	Blade tip	4.18	1/20
3	Tip 2	Blade tip	8.39	2/20
4	Tip 3	Blade tip	12.58	3/20
5	Middle 1	Blade middle	3.51	1/20
6	Middle 2	Blade middle	7.06	2/20
7	Middle 3	Blade middle	10.62	3/20
8	Root 1	Blade root	2.86	1/20
9	Root 2	Blade root	5.82	2/20
10	Root 3	Blade root	8.73	3/20

**Table 3:** Statistical features of the TC signal in time domain.

Time Domain	Equations	Time Domain	Equations
Maximum	$X_{\max}=\max(x_{i}(t))$	RMS	$X_{rms}=\sqrt{\frac{1}{N}\sum_{i=1}^{N}x_{i}^{2}}$
Standard deviation	$\sigma =\sqrt{\frac{1}{N}{\sum_{i=1}^{N}\left(x_{i}-\mu \right)}^{2}}$	Kurtosis	$\beta =\frac{1}{N}\sum_{i=1}^{N}x_{i}^{4}$
Root amplitude	$X_{r}={\left(\sum_{i=1}^{N}\sqrt{\left|x\right|}\right)}^{2}$	Peak-to peak	$\max(x_{i}(t))-\min(x_{i}(t))$
Mean value	$\bar{x}=\frac{1}{N}\sum_{i=1}^{N}X(t)$		

**Table 4:** Parameters of the CNN model.

No.	Layer Type	Kernel Size/Stride	Number of Feature Maps	Output Size
1	Input	1 × 1	2048	/
2	Convolution 1	64 × 1/8 × 1	16	256 × 16
3	Pooling 1	2 × 1/2 × 1	16	128 × 16
4	Convolution 2	3 × 1/1 × 1	32	128 × 32
5	Pooling 2	2 × 1/2 × 1	32	64 × 32
6	Convolution 3	3 × 1/1 × 1	64	64 × 64
7	Pooling 3	2 × 1/2 × 1	64	32 × 64
8	Convolution 4	3 × 1/1 × 1	128	32 × 128
9	Pooling 4	2 × 1/2 × 1	128	16 × 128
10	Convolution 5	3 × 1/1 × 1	128	16 × 128
11	Pooling 5	2 × 1/2 × 1	128	8 × 128
12	Convolution 6	3 × 1/1 × 1	128	8 × 128
13	Pooling 6	2 × 1/2 × 1	128	4 × 128
14	Full-connected	512	1	512 × 1
15	Softmax	16	1	16

**Table 5:** Fault pattern of the rotating blades.

No.	Fault Patterns	Crack Positions	Crack (mm)	Percentage	Fault Photo
1	N	None	0.0	0	None
2	T1	Blade tip	4.18	1/20	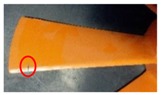
3	T2		8.39	2/20	
4	T3		12.58	3/20	
5	T4		16.73	4/20	
6	T5		20.09	5/20	
7	M1	Blade middle	3.51	1/20	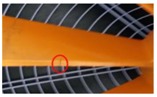
8	M2		7.06	2/20	
9	M3		10.62	3/20	
10	M4		14.22	4/20	
11	M5		17.73	5/20	
12	R1	Blade root	2.86	1/20	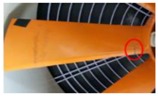
13	R2		5.82	2/20	
14	R3		8.73	3/20	
15	R4		11.62	4/20	
16	R5		14.53	5/20	

**Table 6:** Performance of different classifiers.

Classifiers	SVM	BP	Softmax	FNN
DR (%)	94.5	86.1	91.5	89.5
FAR (%)	9.0	29.0	12.0	12.0
cc (%)	95.1	89.7	91.2	90.8
TrD (s)	28.312	104.726	36.214	63.152
TeD (s)	1.334	4.65	1.733	1.968

**Table 7:** Experimental results.

Predicted Class																	
		N	T1	T2	T3	T4	T5	M1	M2	M3	M4	M5	R1	R2	R3	R4	R5
**Actual class**	**N**	91	6	2	0	0	0	1	0	0	0	0	0	0	0	0	0
	**T1**	7	89	2	1	0	0	1	0	0	0	0	0	0	0	0	0
	**T2**	2	3	91	1	0	0	2	0	0	0	0	1	0	0	0	0
	**T3**	0	1	4	92	2	0	1	1	0	0	0	0	1	0	0	0
	**T4**	0	1	1	2	92	2	0	0	1	0	0	1	0	0	0	0
	**T5**	1	0	0	1	3	93	0	2	0	0	0	0	0	0	1	0
	**M1**	1	0	2	0	1	0	91	1	0	1	0	0	2	0	1	0
	**M2**	0	0	0	2	0	1	2	92	1	0	0	0	1	1	0	0
	**M3**	0	0	0	0	0	1	1	3	94	0	1	0	0	0	0	0
	**M4**	0	0	0	0	0	1	0	0	2	94	1	0	2	0	0	0
	**M5**	0	0	0	0	1	0	0	0	0	2	95	0	0	2	0	0
	**R1**	0	0	0	0	0	1	0	0	0	2	1	94	1	0	1	0
	**R2**	0	0	0	0	0	1	0	0	0	0	2	1	94	1	0	1
	**R3**	0	0	0	0	1	0	0	0	0	0	1	0	1	96	1	0
	**R4**	0	0	0	0	0	0	0	0	0	0	0	0	1	0	98	1
	**R5**	0	0	0	0	0	0	0	0	0	0	0	0	0	0	0	100
